# Comparative Study of Different Methods for Soot Sensing and Filter Monitoring in Diesel Exhausts

**DOI:** 10.3390/s17020400

**Published:** 2017-02-18

**Authors:** Markus Feulner, Gunter Hagen, Kathrin Hottner, Sabrina Redel, Andreas Müller, Ralf Moos

**Affiliations:** Bayreuth Engine Research Center (BERC), Department of Functional Materials, University of Bayreuth, 95447 Bayreuth, Germany; funktionsmaterialien@uni-bayreuth.de (M.F.); funktionsmaterialien@uni-bayreuth.de (G.H.); funktionsmaterialien@uni-bayreuth.de (K.H.); funktionsmaterialien@uni-bayreuth.de (S.R.); funktionsmaterialien@uni-bayreuth.de (A.M.)

**Keywords:** particulate matter, soot, DPF, microwave, cavity perturbation, filter monitoring, soot load determination, resistive soot sensor, integrating sensor, dosimeter

## Abstract

Due to increasingly tighter emission limits for diesel and gasoline engines, especially concerning particulate matter emissions, particulate filters are becoming indispensable devices for exhaust gas after treatment. Thereby, for an efficient engine and filter control strategy and a cost-efficient filter design, reliable technologies to determine the soot load of the filters and to measure particulate matter concentrations in the exhaust gas during vehicle operation are highly needed. In this study, different approaches for soot sensing are compared. Measurements were conducted on a dynamometer diesel engine test bench with a diesel particulate filter (DPF). The DPF was monitored by a relatively new microwave-based approach. Simultaneously, a resistive type soot sensor and a Pegasor soot sensing device as a reference system measured the soot concentration exhaust upstream of the DPF. By changing engine parameters, different engine out soot emission rates were set. It was found that the microwave-based signal may not only indicate directly the filter loading, but by a time derivative, the engine out soot emission rate can be deduced. Furthermore, by integrating the measured particulate mass in the exhaust, the soot load of the filter can be determined. In summary, all systems coincide well within certain boundaries and the filter itself can act as a soot sensor.

## 1. Introduction

Diesel particulate filters (DPF) have become essential parts of exhaust gas aftertreatment systems for diesel engines to meet the increasingly tighter emission limits concerning particulate matter [1]. In a so-called wall flow filter, soot particles accumulate in the pores and on the surface of the ceramic walls of the filter monolith. From time to time, the DPF needs to be regenerated, which means the soot has to be burned off in order to prevent clogging. For an efficient regeneration strategy, the actual trapped soot mass must be known at any time. Hence, accurate soot load determination is very important for exhaust gas aftertreatment applications. It is state-of-the-art to determine the soot load of a DPF model-based supported by a differential pressure signal [2]. A more novel approach uses microwaves to monitor directly the soot load. This technique has been introduced by several groups and is considered very promising [3,4,5]. In our own previous studies, the microwave-based filter monitoring was tested under constant engine operation conditions [3,6]. Recently, Sappok et al. showed that the microwave-based system is also suitable for applications under non-steady operation conditions [7].

The first aim of the present study is to prove these results and to confirm that the microwave-based soot detection systems are able to work at different soot load rates. Furthermore, it shall be investigated whether the DPF itself can act as a soot sensor device following the dosimeter principle (also known as integrating or accumulating principle [8]). Therefore, the signal of the filter monitoring systems (microwave-based and differential pressure) must be differentiated with respect to time to determine the “soot loading rate” of the filter, which according to the theory of integrating sensors should correlate with the actual soot mass concentration in the exhaust entering the filter. In this study, different operation points are adjusted for a certain time, and the slope of the signal at each operation point is correlated with the soot mass concentration. Additionally, the results will be compared with a conductometric soot sensor and a commercially available soot nanoparticle measurement device (Pegasor), both placed upstream of the DPF. Thereby, the Pegasor sensing device, which is widely used in dynamometer test benches, serves as a reference system to determine the actual soot mass concentration. The resistive (also denominated as conductometric) soot sensor correlates the soot mass in the exhaust to the electrical current that occurs when soot deposits on the sensor surface or the time span until this current occurs [9,10,11,12,13,14,15,16]. Both the Pegasor sensing device and the resistive soot sensor measure the soot mass concentration in the raw exhaust. It will be examined whether an integration of the signals over time enables to estimate the trapped soot mass inside the filter, and it will be investigated whether the results coincides with the microwave-based system and the differential pressure sensor signal.

## 2. Materials and Methods

### 2.1. Experimental Setup and Procedure

**Figure 1:**
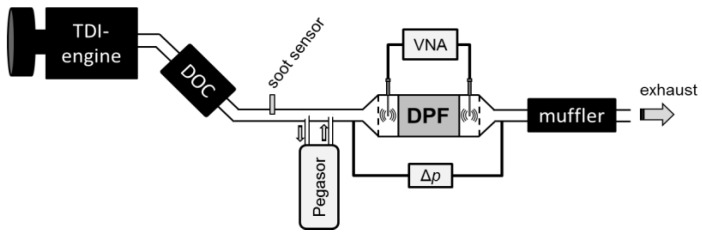
Experimental setup for the dynamometer tests with the different measuring devices: resistive soot sensor and Pegasor sensing device to measure the soot concentration of the raw exhaust, the vector analyzer VNA to obtain the transmission parameter |*S*_21_| in the microwave range between 0.9 and 2.1 GHz and the differential pressure sensor (Δ*p*) for soot load determination. Furthermore, the filter is weighed from time to time.

The tests were carried out in the exhaust of a 4 cyl., 2.1 L turbocharged direct injection (TDI) engine (Mercedes Benz OM651) on a dynamometer test bench. Downstream of an oxidation catalyst (DOC), the diesel particulate filter (DPF), and the different measuring devices were placed in the exhaust pipe. A scheme of the setup can be seen in Figure 1.

The DPF was an uncoated aluminum titanate monolith with a cell density of 300 cells per square inch (cpsi), 5.66″ (14.4 cm) in diameter and a length of 6″ (15.2 cm). The filter was monitored with the microwave-based method. Therefore, two stub antennas were inserted into the filter housing and connected to a vector network analyzer (VNA, MS2025B, Anritsu, Morgan Hill, CA, USA). The VNA recorded the transmission parameters |*S*_21_|, which were then averaged in the frequency range between 0.9 and 2.1 GHz. Further details about this method are described in the next section. Additionally, a differential pressure sensor was installed to measure the pressure drop, Δ*p*, over the filter. These two measurands, Δ*p* and the microwave-derived parameter |*S*_21_|, basically are measures for the accumulation of soot inside the filter. Furthermore, the actual trapped soot mass on the DPF was determined gravimetrically from time to time by dismounting the whole canning and weighing it on a platform scale (DS30K0.1, Kern & Sohn GmbH, Balingen-Frommern, Germany). Thereby it was ensured that the filter was still heated above 150 °C in order to prevent water condensation.

For soot mass determination in the raw exhaust, a custom-built resistive soot sensor was mounted upstream of the DPF. The sensor is briefly described below in Section 2.3 and in more detail in [16]. As a reference system, a commercial Pegasor soot sensing device was used. The operation principle is described in [17,18]. In short, it works as follows: A partial exhaust flow is taken out of the exhaust pipe and guided through the measuring chamber, which basically represents a faraday cage. After charging the contained soot particles electrically by means of a corona, the electrical current is measured when the charged particles leave the cage again. This current correlates with particle number and soot mass.

**Figure 2:**
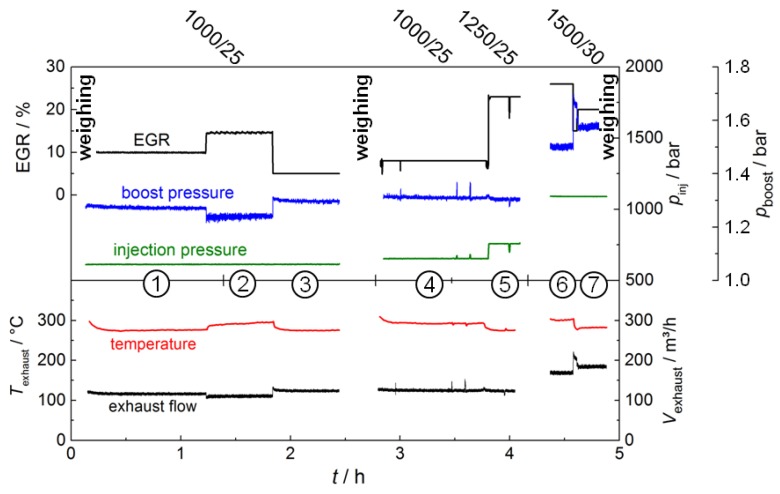
Test procedure: within the seven operation points, different soot concentrations in the exhaust were achieved by varying engine speed, accelerator pedal position, EGR-rate, injection pressure (*p*_injection_), and boost pressure (*p*_boost_). Lower graph: resulting exhaust gas temperature and volume flow during the test.

The test procedure is depicted in Figure 2. In contrast to previous works on microwave-based DPF monitoring, where a constant particle concentration was present in the exhaust [3,6], here, different operation points (①–⑦) with a distinct soot emission rate were achieved by varying several engine parameters. This leads to different soot loading rates of the filter within the test. Parameters to manipulate the combustion process to vary the soot emission were engine speed and accelerator position (see Figure 2: 1000–1500 rpm/25–30%), injection pressure, boost pressure, and exhaust gas recirculation rate (EGR). Before and after the test run, as well as between operation points ③ and ④, the filter was weighed. After operation point ⑤, the engine was deliberately stopped for a short time. This, however, had no effect on the results. The resulting exhaust gas temperature upstream of the DPF and the volume flow (calculated by means of intake airflow and fuel consumption) during the test are shown in the lower graph of Figure 2. The temperature was around 285 °C and varied in a range of ca. 20 °C. The exhaust volume flow ranged between 110 and 125 m^3^/h, besides in the last two operation points ⑥ and ⑦, where the increased engine speed of 1500 rpm evokes a higher exhaust flow rate (170–185 m^3^/h).

### 2.2. Microwave-Based Method for Soot Load Determination

**Figure 3:**
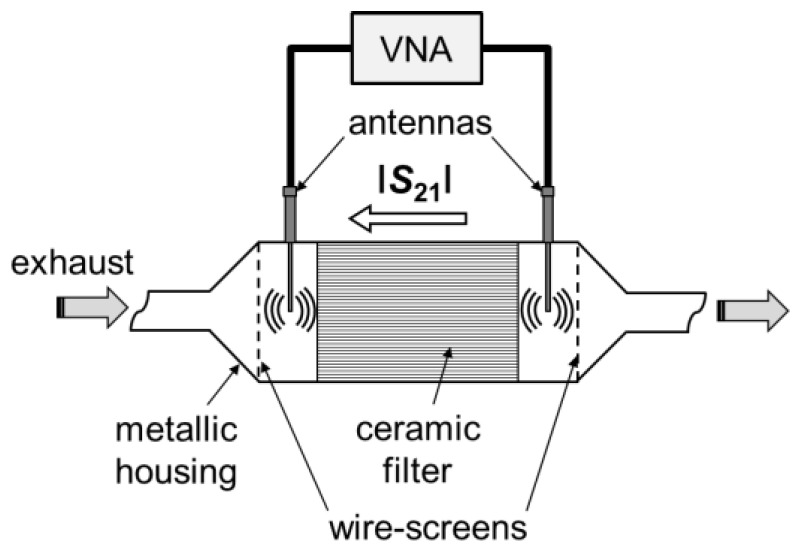
Setup of the microwave-based method for filter monitoring.

The microwave-based method for filter monitoring is described in detail in [3,4,5,6]. In brief, it works as follows: The metallic filter housing acts as a cavity resonator for electromagnetic fields since it is electrically conducting. With a stub antenna, an electric field can be impressed into the housing. A characteristic field distribution, which depends on the resonator geometry and the electric material properties of the dielectric inside the resonator, forms. With the same or with a second stub antenna, the resulting field can be measured. Thereby, the frequency-dependent reflection or transmission parameters (|*S*_11_| or |*S*_21_|, respectively) of the microwaves can be determined. Here, one antenna was used downstream of the catalyst to emit the electromagnetic waves, and a second one upstream of the DPF to measure the transmission, |*S*_21_|, of the microwaves through the DPF (see Figure 3 for a scheme of the setup). The frequency-dependent |*S*_21_| signal was transformed into |*S*_21_|/dB = 20 × log|*S*_21_| and was averaged in a frequency range from 0.9 to 2.1 GHz. The stub antennas were designed in a way that the inner conductor reaches the center of the cylindrical resonator. A vector network analyzer (Anritsu, MS2025B) was used to determine the S-parameters with a sampling rate of 1 min^−1^. The sampling rate could be increased by using faster measuring equipment or measuring in a narrower frequency range. Averaging was performed in a post-processing step and did not influence the sampling rate. In a later (serial) application, post processing could be integrated into a sensor control unit, which may be built up with reduced functionality compared to a full network analyzer. There are already commercial systems available following this principle ([19,20]).

As the ceramic filter element is located inside of the resonator (Figure 3), soot accumulation in the filter affects the field distribution by changing the overall electric properties (especially complex permittivity, *ε*, and conductivity, *σ*); the *S*-parameters are hence affected. Since soot is known to be electrically conductive [21,22], the field strength gets attenuated. This leads to a decrease of the averaged (in a certain frequency range) transmission value |*S*_21_|. This signal correlates well with soot load [6] and hence can be used to monitor the loading degree of the DPF.

At both ends, the canning is electrically closed by wire screens. This is not necessary in real world application, but it defines the resonator geometry more exactly, which is helpful for research purposes since it reduces the number of side modes.

### 2.3. Resistive Soot Sensor

The soot sensor used in this study was a resistive type sensor as described in [16]. It was made in thick-film technology. Basically, this type of sensors comprises two planar interdigital sensing electrodes (line = space = 100 µm) on an insulating substrate. A DC-voltage is applied between them. When soot particles accumulate on the sensor surface, conductive paths form between the electrodes and an electrical current becomes measurable. This current increases the more soot accumulates on the sensor. The slope of the sensor current (time derivative), which serves as the actual sensor signal, correlates well with the soot concentration in the exhaust [16]. The soot accumulation might be influenced by the applied voltage and the sensor temperature [23]. In this study, 20 V DC were applied. The sensor was not heated during soot collection, i.e., it showed approximately exhaust temperature. The first period of the linear current increase was taken to determine the slope. From time to time, the sensor was regenerated by being heated up to about 600 °C with an integrated heater structure. This leads to soot oxidation and afterwards, soot accumulation could start again. Consequently, the response of such sensors does not provide a continuous signal but single measuring points, as the slope of the current of each measuring cycle needs to be determined. In this study, the heater structure on the reverse side as well as the sensing electrodes on the front side were made from Pt (LPA88, Heraeus, Hanau, Germany), screen-printed on an alumina substrate (Rubalit 708S, CeramTec, Plochingen, Germany). The heater and the electrical feed lines were covered by an alumina based dielectric protective layer (QM42, DuPont, Wilmington, DE, USA). After wiring, the sensor was housed in a stainless steel tube and mounted in the exhaust pipe of the engine. The sensor was oriented in a way that the sensing electrodes faced the gas flow directly.

## 3. Results and Discussion

### 3.1. Soot Load Determination

**Figure 4:**
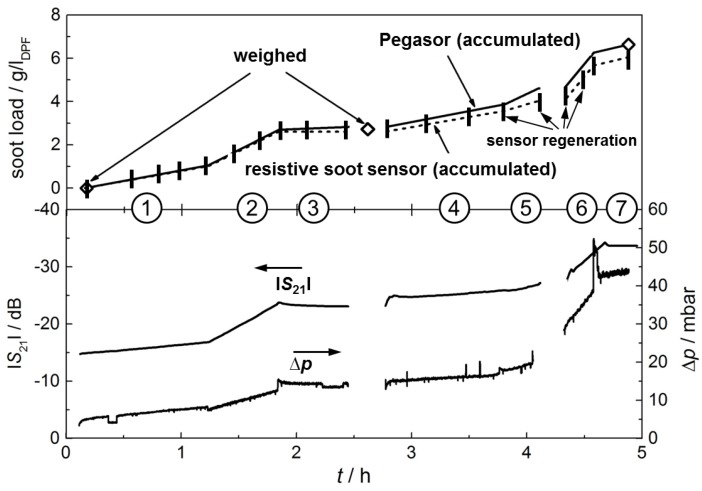
Upper graph: soot load of the DPF, determined by weighing (open squares), with the Pegasor sensing device (solid line, corrected), and with the resistive soot sensor (dashed line, corrected). The vertical bars indicate regeneration events of the resistive soot sensor. Lower graph: microwave-derived transmission parameters, |*S*_21_| in dB, and differential pressure, Δ*p*.

The results of the test are shown in Figure 4. The numerals ① to ⑦ again represent the operation points of the engine. In the upper graph, the weighed soot load is plotted in *g*/*l*_DPF_ (open squares). As described above, the filter was weighed before and after the test run and once in the middle of the test.

The soot concentration as determined by the Pegasor sensing device in g/m^3^ in each operation point was multiplied by the volumetric exhaust flow rate at the particular operation point and then integrated to get a soot mass inside the DPF. This soot mass is then divided by the DPF filter volume to get a soot load of the DPF in *g*/*l*_DPF_ (Equation (1)):

(1) $$\mathrm{soot}\;\mathrm{load}=\;\frac{m_{\mathrm{soot}}}{V_{\mathrm{DPF}}}\;=\;\frac{\int c_{\mathrm{soot}}\cdot {\dot{V}}_{\mathrm{exhaust}}\;dt}{V_{\mathrm{DPF}}}\;.$$

By doing this, it is assumed for simplification that no soot is being oxidized during the test and that all particles being present in the exhaust gas upstream of the DPF are trapped inside the filter. With this method, the gravimetrically determined actual soot load was slightly underestimated (ca. 16%), which is probably due to an insufficient calibration of the Pegasor sensing device. This is why the calculated soot load is divided by a factor of 0.84 (which is the quotient from the soot load at the end of the test determined by the Pegasor sensing device and that determined by weighing) to normalize the soot load with the gravimetrically determined values. Now, the curve of the soot load determined by the Pegasor sensing device represents the actual soot load during the experiment very well. It can be seen that the graph hits the weighed value at 2.6 h exactly, although only the value at the end was taken into account for the correction.

Very clearly, the single operation points with different particle contents in the exhaust can be distinguished in the slope of the curve with respect to the soot load of the DPF (Figure 4, upper graph). Operation points with a higher average soot mass concentration induce faster filter loading. This can be seen, e.g., in the first half of the test, where by varying only the EGR rate, three different points with different soot mass emissions were adjusted. Operation point ② has the highest EGR rate, which leads also to the highest soot particle formation. The slope of the soot load curve is also the highest at this point. As a conclusion, it can be stated that, by integrating the signal of the Pegasor sensing device, a realistic soot load determination is possible, after calibration of the measuring system.

The question whether it is possible to detect the filter loading with a resistive soot sensor shall be investigated next. In Figure 4, the soot load data derived from the resistive sensor are plotted. It was calculated as follows. At each operation point, an average value for the different slope values of the soot sensor current, d*I*/d*t*, was determined and correlated to the average soot concentration at this operation point, measured by the Pegasor sensing device. The signal slope of each soot-collecting period of the sensor was determined manually. In this way, each soot-collecting period evokes one measuring point. In each engine operation point, a varying number of collecting and regeneration cycles of the sensor are conducted (between one and four). The mean value of the slopes in each engine operation point is then used as a measure for the average soot mass concentration. The resistive type soot sensor was regenerated 18 times throughout the test run for a time of always 60–90 s. Thereby, the response time (the time until first soot paths have formed out and a current is measurable) varies from only seconds, up to minutes, depending on the soot content in the exhaust. Regeneration events are indicated in Figure 4 by vertical bars.

**Figure 5:**
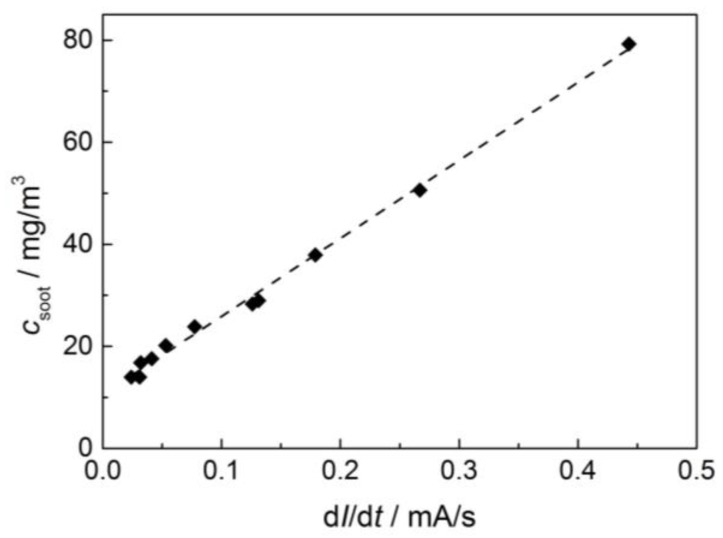
Correlation of the timely differentiated resistive soot sensor signal, d*I*/d*t*, and the soot concentration in the exhaust, *c*_soot_, as determined by the Pegasor sensing device. The dashed regression line follows the equation: *c*_soot_/mg/m^3^ = 152.76·d*I*/d*t*/(mA/s) + 10.6.

Figure 5 shows the very good linear correlation between the sensor signal and the soot mass in the exhaust. Thereby, various test runs are considered for defining the linear regression curve, which are not shown here specifically. With this regression curve (Figure 5), an average soot concentration can be determined for each operation point from the soot sensor signal. Now, this averaged soot mass concentration was again multiplied with the related exhaust flow and integrated over time (Equation (1)). This procedure is similar to the calculation of the DPF soot load from the signal of the Pegasor sensing device. Again, the correction factor of 0.84 was used to calculate the soot load.

The two curves in the upper graph of Figure 4, the soot load as determined by the Pegasor sensing device and the soot load as determined by the resistive soot sensor, agree well. The different average soot mass concentrations in the exhaust of each operation point, which yields different slopes of the soot load curves, can be seen by both systems. However, the soot load, especially after operation point ⑤, is underestimated by the resistive soot sensor.

In the lower graph of Figure 4, the microwave-derived frequency-averaged transmission parameter |*S*_21_| and the differential pressure, ∆*p*, are plotted. The slopes of the curves at each operation point coincide quantitatively very well with the slope of the soot load of the DPF (upper graph). Both curves show a slight decrease in operation point ③, in contrast to the values calculated from the Pegasor sensing device and from the resistive soot sensor. This could most likely be due to soot oxidation inside the filter, resulting in a slightly decreased soot load in the DPF. Yet, in the exhaust gas upstream of the DPF, soot particles are still present and are detected by the resistive soot sensor and the Pegasor sensing device, which are located upstream of the filter. In contrast to that, the differential pressure and the microwave-derived transmission parameter are not affected by the soot mass concentration upstream of the filter, as both correlate to the soot *load* of the filter.

**Figure 6:**
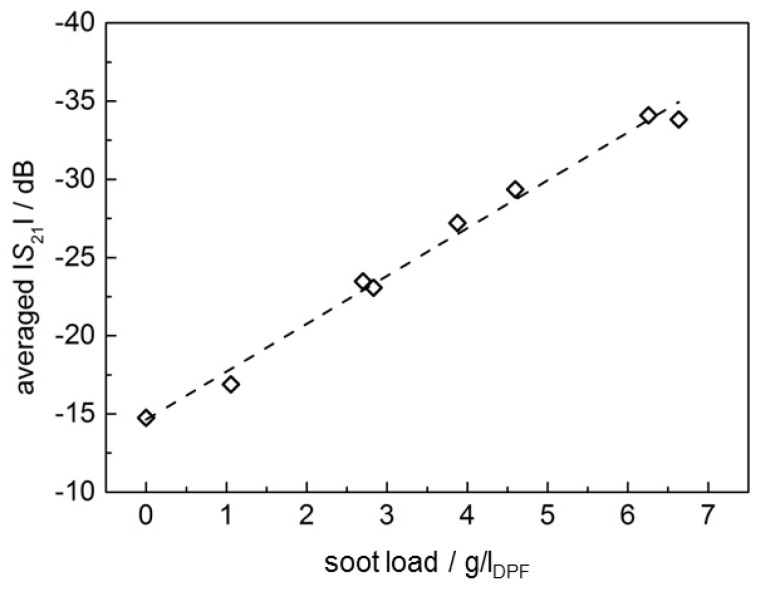
Correlation of the frequency-averaged transmission parameter |*S*_21_|and the soot load of the DPF. The dashed regression line follows the equation: |*S*_21_|/dB = −3.061·*m*_soot_/(*g*/*l*_DPF_) − 14.64.

The transmission parameter |*S*_21_| is averaged over frequency as described above. In Figure 6, the value at the beginning of the test run (0 mg/*l*_DPF_) and the end of each operation point is plotted over the corresponding soot load (which is actually the trapped soot mass), which was taken from the accumulated (and corrected) Pegasor sensing device signal in Figure 4. As can be seen in Figure 6, the averaged |*S*_21_| depends linearly on the soot load of the filter. This confirms earlier results [6]. In contrast to the previous study in [6], the filter here was not soot loaded at one single constant engine operation point, but under changing engine parameters. It should be pointed out that there is no compensation of exhaust temperature, although this is an important noise factor [6]. By compensating the temperature influence, the accuracy could have been enhanced even more.

### 3.2. Soot Mass Concentration in the Exhaust Gas

In the previous section, the signals of two devices that were installed in the exhaust gas upstream of the filter to detect the actual soot mass concentration (Pegasor sensing device and resistive soot sensor) were integrated over time and correlated with the actual soot mass loading of the filter. The correlation between the microwave-based signal and the actual soot mass loading of the filter was also shown.

**Figure 7:**
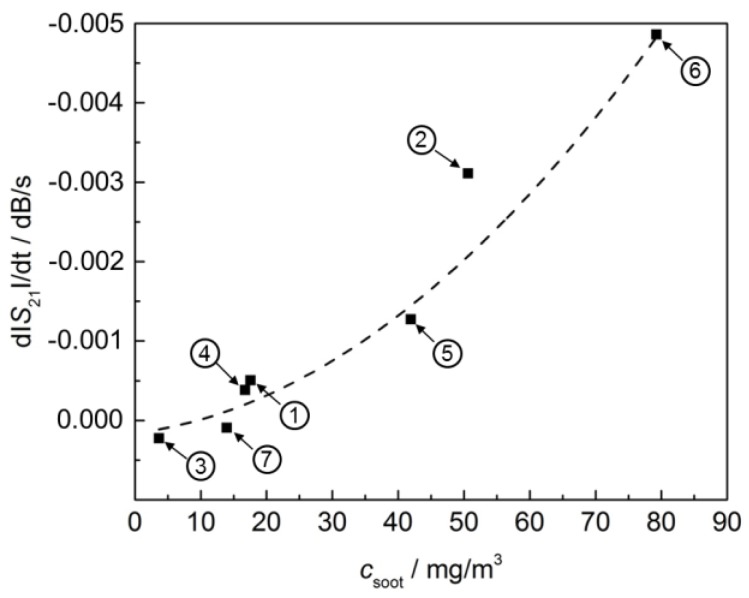
Time derivative of the frequency-averaged transmission parameter |*S*_21_|over the averaged soot mass concentration in the exhaust upstream of the DPF as determined by the Pegasor sensing device. Each point was obtained from one of the operation points ① to ⑦, as indicated in Figure 4. The exponential equation d|*S*_21_|/d*t*/(dB/s) = 0.24138 · exp(−*c*_soot_/(mg/m^3^)/3315.8663) − 0.24062 was used for regression (dashed).

**Figure 8:**
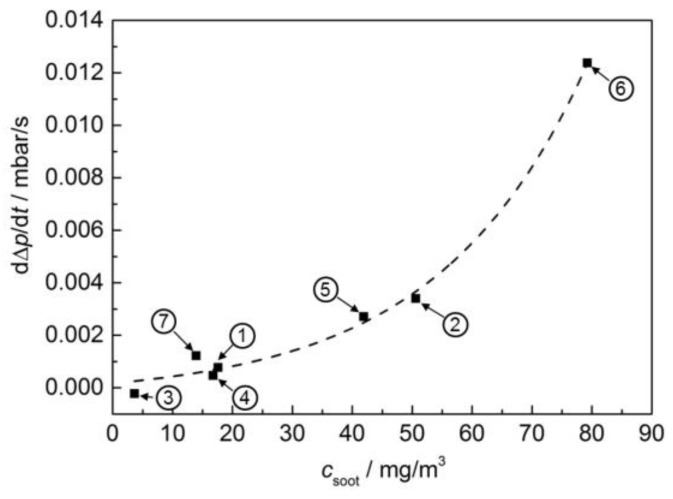
Time derivative of the differential pressure (normalized to exhaust flow) over the averaged soot mass concentration in the exhaust upstream of the DPF as determined by the Pegasor sensing device. Each point was obtained from one of the operation points ① to ⑦, as indicated in Figure 4. The exponential equation d*p*/d*t*/(mbar/s) = 5.29354 × 10^−4^ · exp(−*c*_soot_/(mg/m^3^)/24.93527) − 3.58073 × 10^−4^ was used for regression (dashed).

Now, in contrast to that, it shall be investigated, whether the microwave-based method can be applied to determine the actual soot mass concentration. This has already been described by Sappok et al. in [7]. The results shall be proven here by similar tests with our own system for the first time. Here, the filter itself acts as the soot sensor. If one neglects second order effects, changes in the soot loading of the filter should be proportional to the actual soot mass concentration upstream of the filter. Hence, the time derivative of the soot mass may be a suitable means to monitor the soot concentration. This is similar to the gas dosimeter principle where both the accumulated amount and the instantaneous level can be measured even at low concentrations [24]. In Figure 7, the time derivative of the frequency-averaged transmission parameter |*S*_21_| is plotted against the averaged soot mass concentration in the exhaust gas, measured with the Pegasor sensing device. Each point in Figure 8 is a result from one of the operation points ① to ⑦.

In Figure 8, the time derivative of the differential pressure is plotted against the averaged soot mass concentration in the exhaust upstream of the DPF, measured with the Pegasor sensing device. Thereby the change in differential pressure is corrected to exhaust gas flow of this operation point, as it is well known, that the ∆*p* signal is strongly affected by gas flow. With increasing volumetric gas flow, the differential pressure at constant filter loading increases as well [2].

It can be seen that both signals depend similarly on the averaged soot mass concentration. With more soot being present in the exhaust, the soot load of the filter increases faster and hence the derived signal increases. In case of the microwave-based system, it seems that a certain soot mass concentration is needed to evoke a signal. One point (operation point ② at 50 mg/m^3^) seems to be too high and is not considered for the regression curve (dashed line) in Figure 7. It is also striking that both systems (Figure 7 and Figure 8) indicate a slightly negative average soot mass concentration for the value derived from operation point ③ (very low soot concentration measured by the sensors). This may be due to the fact that both systems do not measure the soot mass concentration in the gas phase, but indirectly via the change of the soot load of the filter. Conditions promoting soot oxidation in the filter (elevated temperature, high NO_x_ concentration) lead to a decrease of the signal below zero, despite the fact that a small amount of soot is emitted from the engine and is present in the exhaust even at these operation points. This leads to inaccuracies, especially at low soot mass concentrations. At higher soot contents in the exhaust, the correlation looks quite well. Here, the systems are suitable not only to monitor the filter state, but also to draw inferences about the soot mass concentration of the exhaust.

**Figure 9:**
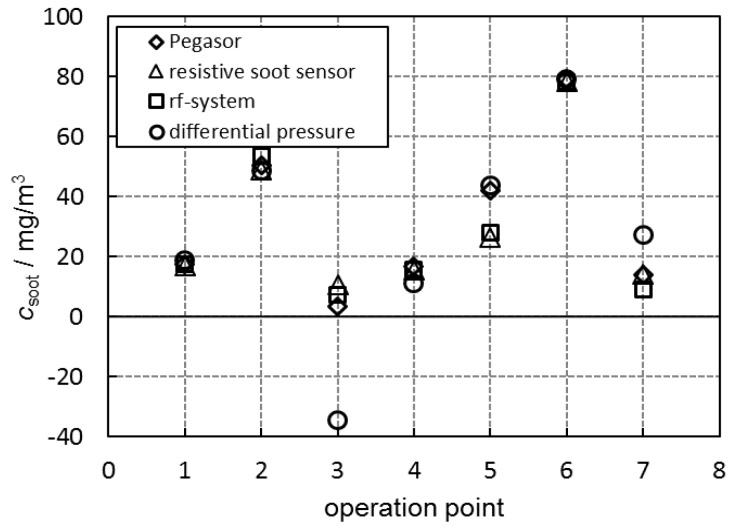
Comparison of all measurement devices in determining the averaged soot mass concentration, *c*_soot_, in the raw exhaust: measured with the Pegasor sensing device (◊), derived from the resistive soot sensor signal (∆), from the time derivative of the averaged microwave-derived transmission |*S*_21_| (□) and from the time derivative of the differential pressure ∆*p* (○).

The before-shown results of the two signals differential pressure and microwave parameter for the averaged soot mass concentration are now compared with the actual soot mass in the exhaust (measured with the Pegasor sensing device) and the signal of the resistive soot sensor. Therefore, all four signals are shown together in Figure 9 for the seven engine operation points. By means of the regression curves (Figure 5, Figure 7 and Figure 8), the soot concentration, *c*_soot_, was calculated for each operation point out of the measured values from the resistive soot sensor, the microwave-based system and the differential pressure, respectively. In Figure 9, the good agreement of the different sensor systems becomes obvious. It has to be noted that temperature correction was neither applied for the microwave-based system nor for the resistive soot sensor. Since exhaust gas temperature varies only around ca. 20 °C in maximum, the influence is not dominant, but temperature compensation may probably be important to increase accuracy. Again, an unrealistic negative value of the differential pressure sensors stands out. At operation point ③, a negative soot concentration of −34 mg/m^3^ was calculated. As described above, this could retrieve from soot oxidation effects or inaccuracies in the measuring procedure, especially at very low soot concentrations. Further, at operation point ⑦, the ∆*p* value diverges compared to the other systems. Apart from this behavior of the differential pressure in some operation points, all measuring devices show consistent soot concentration values. Only in operation point ⑤ do both measuring devices upstream (Pegasor and resistive soot sensor) agree very well, as do both filter sensing devices (microwave-based system and differential pressure sensor). However, it appears that the differentiated filter data do not coincide with the upstream soot concentration data. This has not been understood up to now.

## 4. Conclusions

In summary, it can be stated that all four methods provide consistent results. Soot load determination is possible with the state-of-the-art differential pressure as well as with the microwave-based approach. Thereby, the microwave-based signal is much less affected by side effects as the differential pressure (Δ*p*) sensor. The differential pressure strongly depends on exhaust flow and exhaust temperature (as the volumetric flow is a function of temperature) [2], whereas in case of the microwave system only the temperature needs to be corrected for a reliable measurement [6]. In the here-shown study, even without this compensation, the microwave-based system exhibits a very good linear dependency on the soot load of the filter. For the first time, a filter loading under non-constant engine parameters and hence changing amount of soot mass concentration in the exhaust has been conducted and monitored with our own microwave-based system. These data support the study of Sappok et al. in [7]. The signal follows the measured and expected soot load rates very well. Furthermore, accuracy could even be increased by conducting a temperature correction. By integrating the measured average soot mass concentration, a soot load determination of the filter is also possible with a sensor placed upstream of the filter, like a resistive soot sensor or a Pegasor sensing device. The resistive type sensor would be a good tool for on board raw exhaust soot concentration measurements, whereas the much more precise Pegasor sensing device is not suitable for operation in real vehicles due to its size and due to the expensive required measurement equipment. Note that, if placed upstream of the filter, like here, it is not the aim of a soot sensor to be used for On-Board Diagnosis (OBD) to detect filter malfunction. OBD is an important topic and required by law, but requirements for an OBD sensor downstream of the filter are different, as for example the soot mass concentration values are by far smaller than in our application upstream of the DPF.

However, there are some disadvantages when monitoring a DPF with a sensor upstream of the filter. For instance, soot oxidation inside the filter cannot be detected, leading to lower accuracy in the soot mass determination. Therefore, the sensor-based calculation should be supported, e.g., by a model, considering engine and exhaust parameters like NO_x_ emissions and exhaust temperature.

Seen from the other side, the soot mass concentration of the exhaust upstream of a DPF can be estimated by mathematical derivation of the signals of filter monitoring systems. Both the first time derivative of the differential pressure signal as well as of the averaged |*S*_21_| show a good correlation with the averaged soot mass concentration. In case of a very low amount of soot in the exhaust, this indirect method may not be very accurate, but for higher soot mass concentrations, the monitored DPF itself can practically serve as a soot sensor. In further tests, real-world-like transient conditions should be tested. The here-shown results have been achieved under variable engine operation conditions, but the timely changes were relatively slow and each operation point was held constant for a couple of minutes. The performance of the measuring systems during more realistic transient engine operation is not reflected here. For such transient tests, signal post processing would have to be adjusted, as the time period for the mathematical operations (averaging, integration, and derivation) must be reduced to perform faster measurements. For an application, the influence of stored ash in the filter and, if necessary, other side effects must be considered. For the here-shown tests, stored ash is not a problem, as the amount of ash in the filter is practically not changing during the test. However, throughout the lifetime of a filter in a real application, ash accumulates inside the filter, influences both the differential pressure and the signals of the microwave-based system. This problem, however, could be solved by calibration, e.g., after a complete active filter regeneration. This needs to be solved anyway by application of all systems for soot load determination. In this study, it should only be proven whether it is basically possible to use the filter itself as a soot sensor, so as to present an “additional benefit” that is obtained without further sensing devices if one applies the microwave-based system for soot load determination anyway, as it is shown in the literature. Considering the cost of the microwave-based system, it has to be noted that the sampling device is not very complex. The antennas basically represent just a steel rod, coaxially placed in a steel tube. As mentioned above, a full VNA will not be required for application, as not all the functions provided by a VNA are really needed. Instead, a sensor control unit that consists of inexpensive components from mobile phone technology could be built. Inexpensive devices have already been presented [19,20].

As in our previous work, the resistive soot sensor again showed a very good correlation with soot mass concentration.
